# Physicochemical and Nutritional Requirements for Axenic Replication Suggest Physiological Basis for *Coxiella burnetii* Niche Restriction

**DOI:** 10.3389/fcimb.2017.00190

**Published:** 2017-05-31

**Authors:** Eduardo Vallejo Esquerra, Hong Yang, Savannah E. Sanchez, Anders Omsland

**Affiliations:** Paul G. Allen School for Global Animal Health, College of Veterinary Medicine, Washington State UniversityPullman, WA, United States

**Keywords:** obligate intracellular parasite, niche restriction, *Coxiella*, gluconeogenesis, axenic culture

## Abstract

Bacterial obligate intracellular parasites are clinically significant animal and human pathogens. Central to the biology of these organisms is their level of adaptation to intracellular replication niches associated with physicochemical and nutritional constraints. While most bacterial pathogens can adapt to a wide range of environments, severe niche restriction—an inability to thrive in diverse environments—is a hallmark of bacterial obligate intracellular parasites. Herein the physicochemical and nutritional factors underlying the physiological basis for niche restriction in the zoonotic bacterial obligate intracellular parasite and Q fever agent *Coxiella burnetii* are characterized. Additionally, these factors are reviewed in the context of *C. burnetii* evolution and continued (patho) adaptation. *C. burnetii* replication was strictly dependent on a combination of moderately acidic pH, reduced oxygen tension, and presence of carbon dioxide. Of macronutrients, amino acids alone support replication under physicochemically favorable conditions. In addition to utilizing gluconeogenic substrates for replication, *C. burnetii* can also utilize glucose to generate biomass. A mutant with a disruption in the gene *pckA*, encoding phosphoenolpyruvate carboxykinase (PEPCK), the first committed step in gluconeogenesis, could be complemented chemically by the addition of glucose. Disruption of *pckA* resulted in a moderate glucose-dependent growth defect during infection of cultured host cells. Although, *C. burnetii* has the theoretical capacity to synthesize essential core metabolites via glycolysis and gluconeogenesis, amino acid auxotrophy essentially restricts *C. burnetii* replication to a niche providing ample access to amino acids. Overall, the described combination of physiochemical and nutritional growth requirements are strong indicators for why *C. burnetii* favors an acidified phagolysosome-derived vacuole in respiring tissue for replication.

## Introduction

Bacterial obligate intracellular parasites represent some of the most clinically significant pathogens, including the sexually transmitted bacterium *Chlamydia trachomatis* (Jeanne Marrazzo, 2014), and the agent of epidemic typhus, *Rickettsia prowazekii* (Bechah et al., 2008). Central to the biology of this group of organisms is their adaptation to replicate within specific intracellular niches. Additionally, many bacterial obligate intracellular parasites have undergone reductive evolution, resulting in relatively small genomes of ~1–2 Mega bases (Mb) that code for fewer than 2,000 genes (Andersson et al., 1998; Kalman et al., 1998; Seshadri et al., 2003). For the pathogen, this form of genome streamlining comes with a cost: reduced metabolic capacity and physiological plasticity that is necessary to adapt to diverse environments. Hence, a hallmark characteristic of bacterial obligate intracellular parasites is niche restriction, the inability to thrive in diverse environments.

The intimate association between bacterial obligate intracellular parasites and their host cells dictates that alterations in host cell physiology may affect fitness and replication of the parasite. However, lack of host cell-free (axenic) culture tools to study bacterial intracellular parasites prevents analysis of the physicochemical and nutritional determinants required for replication. Consequently, the parameters underlying niche restriction for specific bacterial obligate intracellular parasites are largely unknown.

*Coxiella burnetii* is a zoonotic bacterial obligate intracellular pathogen and the cause of Q (query) fever. In humans, Q fever manifests as a debilitating illness with influenza-like symptoms that can progress to potentially fatal Q fever endocarditis (Maurin and Raoult, 1999). Following infection of cultured cells, *C. burnetii* replicates in a vacuole within the host cell cytoplasm referred to as the *Coxiella* Containing Vacuole (CCV) (Voth and Heinzen, 2007; Kohler and Roy, 2015). The pathogen's life cycle follows a characteristic bi-phasic developmental program that includes developmental transitions between two distinct cells forms, the replicative Large Cell Variant (LCV) and the non-replicative Small Cell Variant (SCV) that accumulates in stationary phase (Coleman et al., 2004). Demonstrated activity of acid phosphatase and 5′-nucleotidase (Burton et al., 1978, 2011) within the CCV indicates a phagolysosomal origin of the vacuole. This evidence motivated studies that showed *C. burnetii* metabolite transport and catabolism, as well as intracellular replication, are strictly dependent on a moderately acidic environment (Hackstadt and Williams, 1981a). Although, an intracellular parasite under natural conditions, *C. burnetii* is culturable using specially designed axenic media (Omsland et al., 2009, 2011; Omsland, 2012; Sandoz et al., 2016). Axenic culture allows for separation of *C. burnetii* from its host cell, providing conditions to dissect the organism's growth requirements.

Here, we exploit axenic culture of *C. burnetii* to identify physiologically relevant physicochemical and nutritional requirements for replication. *C. burnetii* replication was strictly dependent on a combination of moderately acidic pH, reduced oxygen tension and presence of carbon dioxide. Provided amino acid auxotrophy is satisfied, *C. burnetii* can utilize both glycolysis and gluconeogenesis to support biomass generation. These findings can explain the physiological basis for the obligate intracellular nature of *C. burnetii*, thus defining key parameters of *Coxiella* niche restriction.

## Materials and methods

### Bacteria and culture conditions

*C. burnetii* Nine Mile phase II clone 4 (RSA 439) has been described elsewhere (Howe et al., 2010). Nutrient media used for cultivation of *C. burnetii* included ACCM-2 (Omsland et al., 2011), and Defined Acidified Citrate Medium (D-ACM) (13.4 mM citric acid, 16.1 mM tribasic sodium citrate, 3.7 mM potassium phosphate, 1 mM magnesium chloride, 0.09 mM calcium chloride, 10 μM iron sulfate, 124.7 mM sodium chloride, 1 mg/ml methyl-β-cyclodextrin, and individual amino acids at 1.5 mM, pH 4.75) (Table S1). The following amino acids were dissolved at a concentration of 3 mM in dH_2_O: methionine, arginine, glycine, cysteine, proline, lysine, and isoleucine. The following amino acids were dissolved at a concentration of 3 mM in a 2X solution of the acidic basal buffer of D-ACM: alanine, valine, asparagine, threonine, histidine, phenylalanine, leucine, tryptophan, and glutamic acid. Tyrosine was dissolved in a small volume of 1 N HCl. Liquid cultures were established in T-75 or T-25 cell culture flasks containing 20 or 7 mls medium, and inoculated with 10^6^ CFUs per ml medium. Sub-culturing tests were done with the D-ACM starter culture in mid log phase using a 1:1,000 dilution to inoculate the sub-culture. D-ACM used for sub-culture tests was prepared using analytical standard grade amino acids to reduce the likelihood of micronutrient (e.g., vitamins) contamination. Solid media were prepared by mixing a sterile filtered 2X preparation of the buffer and amino acids (pH 4.5) with an equal volume of sterile 0.5% (w/vol. in dH_2_O) high gelling temp agarose (Fisher BioReagents). Plates were prepared with 20 ml medium per plate. Liquid media were sterile filtered. *C. burnetii* stock CFU titer was determined by plating on solid (final 0.25% w/vol. agarose) ACCM-2 supplemented with 500 μM tryptophan, pH 4.5 (Figure S1). Unless otherwise noted, *C*. *burnetii* was incubated at 37°C under conditions of 5% CO_2_, and 5% O_2_ (air was replaced by N_2_) using MCO tri-gas incubators (Panasonic Healthcare Co.). *C. burnetii* was stored at −80°C in ACCM-2 supplemented with 10% DMSO as a cryo-protectant.

### Host cell culture and infection

African green monkey kidney epithelial cells (Vero), were established in RPMI 1640 containing Glutamax and additionally supplemented with 10% heat-inactivated Fetal Bovine Serum (FBS). For verification of infectivity following culture in D-ACM, Vero cell cultures in 6-well plates were infected with the inoculum suspended in 1 ml medium without FBS for 2 h at RT with gentle rocking. Following the 2 h infection period the inoculum was removed and replaced with medium containing 2% heat-inactivated FBS. To test the effect of *pckA* deletion on intracellular replication, Vero cells were established in 12-well plates using the described medium, then maintained during infection using (glucose-free) RPMI 1640 supplemented with 11 (standard concentration) or 5.0 mM glucose, and 2% heat-inactivated FBS from the time of infection. Infections were facilitated by centrifugation (30 min, 400 × g) using an MOI of 5 genome equivalents (GE). Mutant strains were incubated under antibiotic selection as described below. Media were replaced every 24 h to maintain host viability as necessary under glucose limitation. The number of Vero cells recovered from cultures maintained under different glucose availability were not significantly different after the 5 d incubation period (data not shown). Vero cells were incubated at 37°C with 5% CO_2_ using a Steri-Cycle CO_2_ incubator (Thermo Scientific).

### Construction and characterization of *Cb*Δ*pckA*

A *C. burnetii* mutant (*Cb*Δ*pckA*) containing a kanamycin resistance cassette inserted in the gene *pckA* (CBU2092), encoding phosphoenolpyruvate carboxykinase, was generated largely as described (Omsland et al., 2011; Beare et al., 2012). Briefly, primers pckA-5′pJC-F 5′-CGGTACCCGGGGATCCCTGCGCTGCATACGGGCTACTATGAA-3′ and pckA-5′pJC-R 5′-GCACCACCGGTCGACGTCGCCCATAATTTGCTCCATAGCTGTTCC-3′, and pckA-3′pJC-F 5′-CGTCGACCGGTGGTGCCGAGGGTCCATTAGCGAAAATATTAAAAGAAT-3′ and pckA-3′pJC-R 5′-GAACCTGTTTGTCGACGGACGATGGGCGAGAAATCAAATCCTGC-3′ were used to amplify ~ 2.5 kb of genomic DNA upstream and downstream of CBU2092. The underlined nucleotides indicate *Bam*HI, *Sal*I, and *Age*I restriction sites. The flanking sequences were ligated to the linearized suicide plasmid pJC-CAT (Beare et al., 2012) between the *Bam*HI and *Sal*I restriction sites using an In-Fusion HD cloning kit (Clontech Laboratories, Inc.), generating pJC-3′5′CbΔpckA. A kanamycin resistance cassette was amplified from pJB-KAN (Omsland et al., 2011) using primers P1169-Kan-F 5′-GGCGACGTCGACCGGTATGGCTTCGTTTCGCAGCGAACTTGG-3′ and P1169-Kan-R 5′-CCTCGGCACCACCGGTTTATCAGAAGAACTCGTCAAGAAGGC-3′, and then cloned into pJC-3′5′CbΔ*pckA* at the *Age*I restriction site, creating pJC-CbΔpckA. pJC-CbΔpckA was used to transform *C. burnetii* by electroporation essentially as described (Beare et al., 2012). Transformants were expanded in ACCM-2 under kanamycin (350 μg/ml) and chloramphenicol (3 μg/ml) selection. Primary intergrants were isolated by colony extraction after plating on solid ACCM-2 supplemented with antibiotics, then sub-cultured in ACCM-2 supplemented with kanamycin and 1% sucrose for counter-selection. Following counter-selection, a kanamycin resistant *Cb*Δ*pckA* clone was isolated by colony selection, then expanded in liquid ACCM-2 under antibiotic selection. The isolated *Cb*Δ*pckA* mutant was characterized by PCR amplification of the mutated region using primers pckA-out-F 5′-ACCATAATGACGCTAAGTATGCGCGGATC-3′ and pckA-out-R 5′-GAGATTGCGATTTACTCACAGCTAAAAGCG-3′, and by chemical and genetic complementation (**Figure 4** and Figure S2). Genetic complementation in Trans was done using a modified pJB-CAT vector containing *pckA* driven by the promoter 1169^P^ (Omsland et al., 2011). Complemented strains were cultivated in the presence of antibiotics.

### Measurement of culture yield and bacterial genome equivalents

Culture optical density was measured at 600 nm (OD_600_) using a DU-530 spectrophotometer (Beckman). Non-inoculated yet incubated medium was used as a control for the measurements. *C. burnetii* GE were determined were determined essentially as described (Brennan and Samuel, 2003) using a DyNAmo Flash SYBR Green qPCR Kit (Thermo Fisher) and a CFX96 real time PCR Detection System (Biorad). The following primer pair was used for detection of the *C. burnetii dot*A gene: F-5′-GACCCTACTGTCAATGGCAA-3′; R-5′-GGGATGAGGGTTAGCAGTGT-3′.

### Microscopy and image processing

Vero cells infected with mCherry-expressing *C. burnetii* were fixed with methanol following 5 days of incubation and micrographs captured using an EVOS-fl microscope (Thermo Fisher Scientific). For analysis of bacterial ultrastructure, *C. burnetii* was cultivated in D-ACM until mid-logarithmic (7 d) or early stationary (12 d) phases, pelleted by centrifugation, and washed once with D-ACM buffer (no nutrients), pH 4.75. Following a second centrifugation and removal of the wash buffer, the bacterial pellets were re-suspended and fixed (2% paraformaldehyde, 2% glutaraldehyde, 0.1M cacodylate buffer, pH 7.2). Following fixation, samples were rinsed and dehydrated before embedding with Spurr's resin. Ultra-thin sections (70–100 nm) were prepared with an ultramicrotome (Reichert Ultracut R; Leica) and placed on formvar-coated slot grids. Samples were stained with 2% uranyl acetate and post-stained with Reynolds lead citrate. The sections were imaged using a FEI Tecnai G2 transmission electron microscope (FEI Company).

Electron micrographs and images of *C. burnetii*-infected host cells were processed using ImageJ (National Institutes of Health). Changes to contrast and signal intensity were applied to the entire image.

### Statistical analysis

Statistical analyses were done using Prism software (GraphPad Software Inc.).

## Results

### *C. burnetii* exhibits strict physicochemical growth requirements

**Figure 1:**
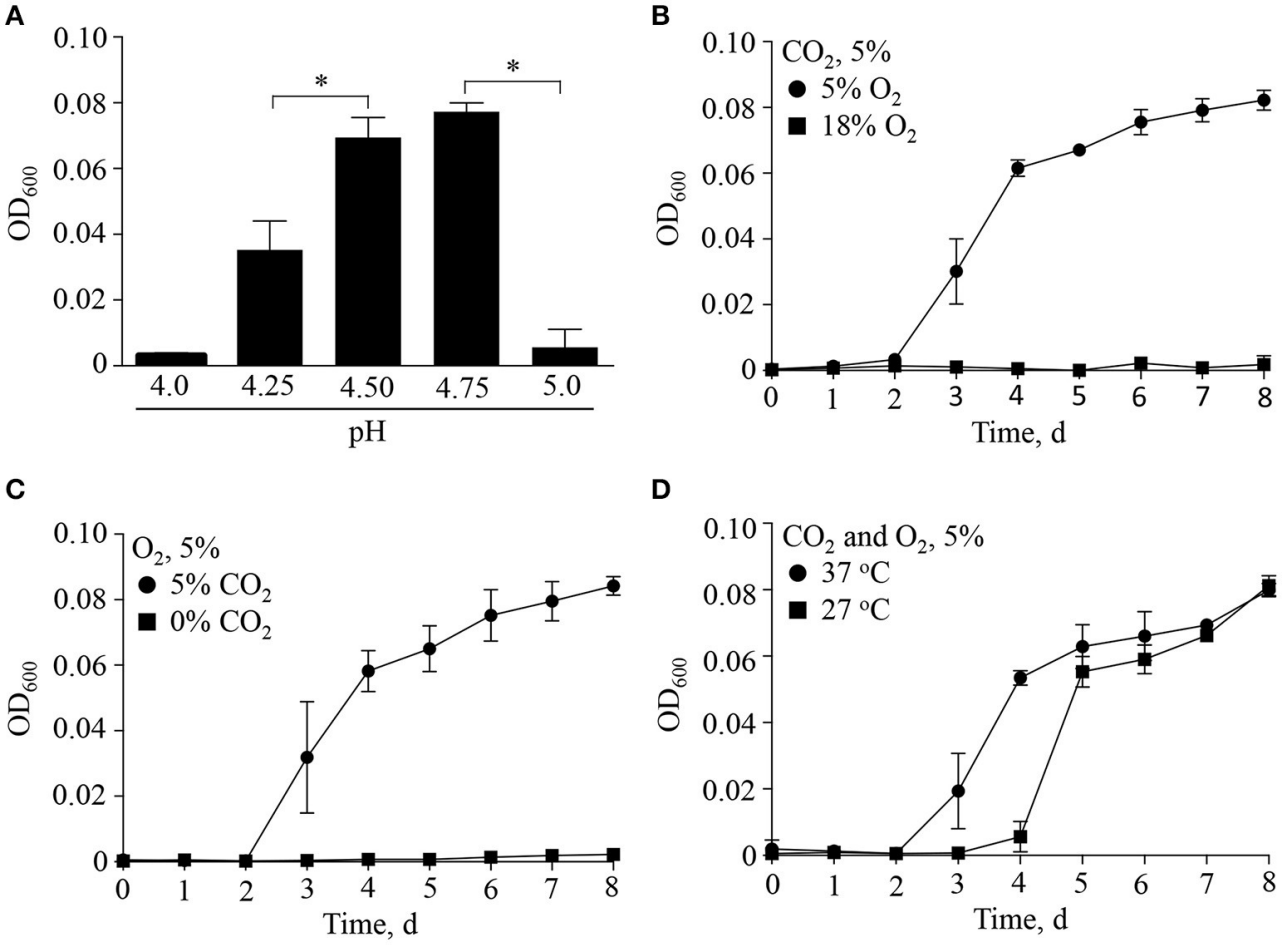
**Multifactorial physicochemical requirements for ***C. burnetii*** replication**. The ability of *C. burnetii* to replicate with alteration of major physiologically relevant physicochemical variables was tested by incubating *C. burnetii* in ACCM-2 under indicated conditions over 8 days. **(A)** Under standard culture conditions at 37°C in an atmosphere of 5% O_2_ and 5% CO_2_ the pH optimum for replication was between 4.5 and 4.75. Under optimal pH conditions, a combination of reduced O_2_ **(B)** and elevated CO_2_ **(C)** was necessary for replication. **(D)** Under conditions of optimal pH, O_2_, and CO_2_, a reduction in culture temperature from 37° to 27°C resulted in an extended lag phase. Data are presented as the mean ± SD (*n* = 3), asterisk indicates statistical significance (ANOVA, Sidak's post-test, *P* < 0.05).

Discovered by Hackstadt and Williams (Hackstadt and Williams, 1981a), *C. burnetii* is a moderate acidophile requiring a pH of ~ 4.5 for optimal metabolite transport, maintenance of a proton motive force (Hackstadt, 1983) and ATP pools (Hackstadt and Williams, 1981b). *C. burnetii* protein synthesis under host cell-free conditions is similarly dependent on moderately acidic pH (Omsland et al., 2008), as demonstrated for both native lysosomes of macrophages (Ohkuma and Poole, 1978) and the CCV (Heinzen et al., 1996; Grieshaber et al., 2002). To determine the permissive pH range for growth of *C. burnetii*, bacteria were incubated in the host cell-free nutrient medium ACCM-2 (Omsland et al., 2011) adjusted to different acidity and replication was measured by optical density (Figure 1). As expected, *C. burnetii* exhibited strict dependence on moderately acidic pH, with optimal growth yields at pH 4.5–4.75. Sharp declines in growth within 0.25 pH units on either side of the optimum were observed (Figure 1A).

In addition to moderately acidic pH, knowledge of *C. burnetii* replication within mammalian tissues suggests other specific factors shape *C. burnetii* niche adaptation. *C. burnetii* typically colonizes the deep tissues of infected animals, including the spleen and liver (Russell-Lodrigue et al., 2006), which represent microaerobic environments (Brooks et al., 2004). As shown previously (Omsland et al., 2009), *C. burnetii* replication under host cell-free conditions is dependent on a reduced oxygen environment. Because the process of respiration not only consumes O_2_ but also produces CO_2_, the dependency of *C. burnetii* replication on a combination of reduced O_2_ and elevated CO_2_ was tested (Figure 1). Indeed, under conditions of 5 or 18% O_2_, and 0 or 5% CO_2_, a combination of reduced O_2_ and elevated CO_2_ tension was necessary for replication (Figures 1B,C). *C. burnetii* yields were equivalent at a CO_2_ tension of 1% to those observed when cultivated under 5% CO_2_ (data not shown). Lastly, because *C. burnetii* infects a wide range of organisms associated with different internal temperatures (e.g., mammals and insects), the effect of temperature on *C. burnetii* replication was tested (Figure 1). A reduction in culture temperature from 37 to 27°C resulted in an approximate 2-day extension of the lag phase but final yield was not affected. These results are consistent with the ability of *C. burnetii* to adjust to vertebrate vs. invertebrate internal temperatures, which is potentially relevant for vector-colonization. Overall, *C. burnetii* appears optimally adapted to the physicochemical characteristics of moderately acidic environments within respiring tissues, where oxygen is depleted and carbon dioxide is generated. Optimal replication was observed at 37°C.

### *C. burnetii* can undergo both glycolysis and gluconeogenesis

The predicted absence of metabolic pathways associated with synthesis of several amino acids (Seshadri et al., 2003) in *C. burnetii* suggests a requirement of the pathogen to scavenge amino acids from its host. *Legionella pneumophila*, a pathogen phylogenetically related to *C. burnetii*, has been shown to stimulate elevation of host amino acid pools upon infection of cultured cells (Price et al., 2011). *Coxiella*-dependent up-regulation of host autophagic activity (Gutierrez and Colombo, 2005; Gutierrez et al., 2005) appears to similarly connect *C. burnetii* to manipulation of a process that stimulates generation of free amino acids.

**Figure 2:**
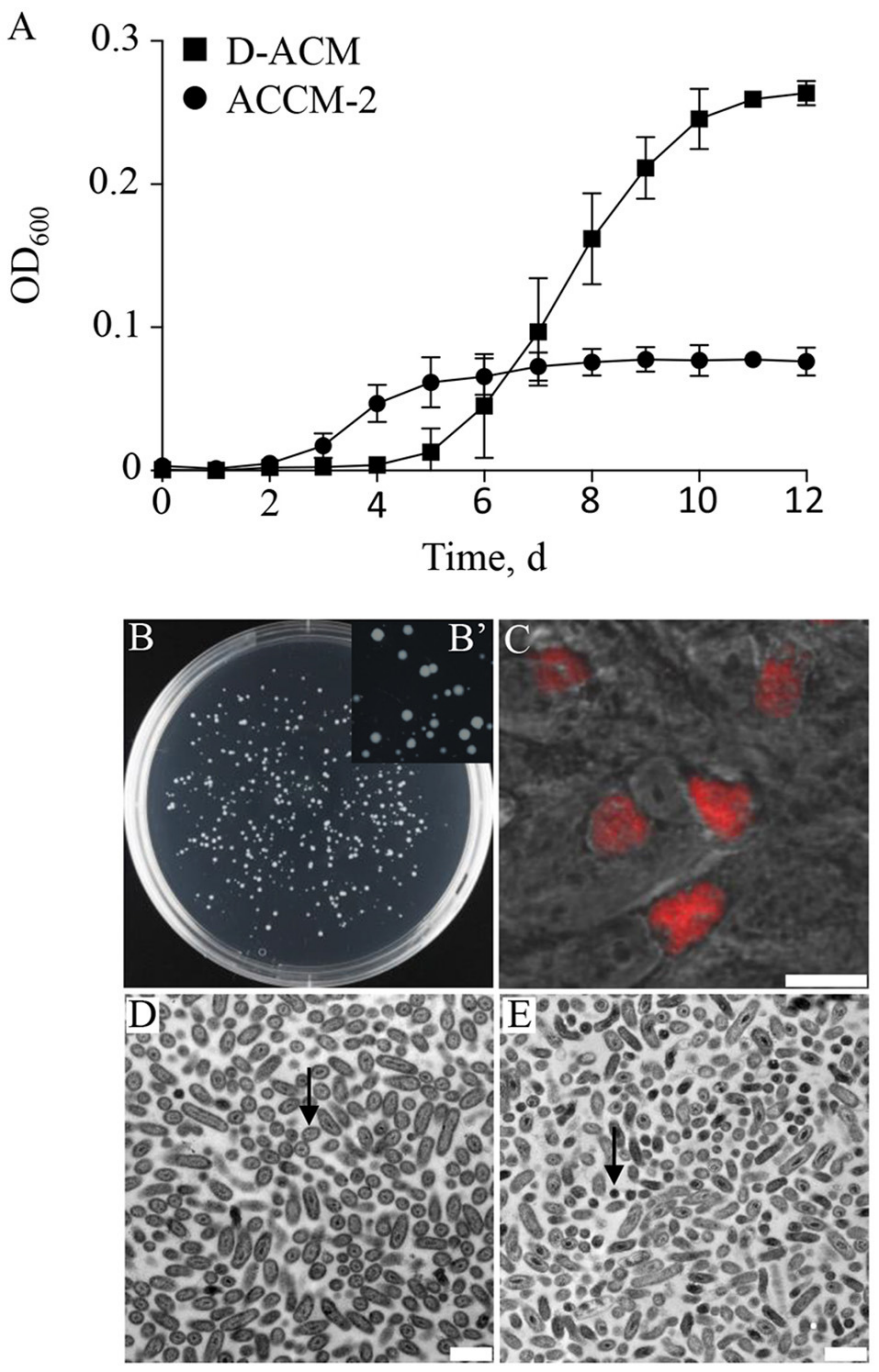
*****C. burnetii*** replication with amino acids as the only available macronutrients**. The ability of *C. burnetii* to generate infectious progeny in a nutritionally limited medium containing 17 amino acids was tested. **(A)** Compared to growth ACCM-2, D-ACM supported ~ 2.5-fold higher yields of *C. burnetii* over 12 days. **(B,B')** Solid D-ACM supported formation of *C. burnetii* colonies following expansion in liquid D-ACM. **(C)** D-ACM supported generation of infectious *C. burnetii* progeny as determined by microscopic verification of CCV development in Vero cells infected with *C. burnetii* expressing mCherry. **(D,E)** *C. burnetii* was cultivated in D-ACM and the ultrastructure of mid-logarithmic **(D)** or early stationary **(E)** phase bacteria analyzed by Transmission Electron Microscopy. The majority of cells observed in the two growth phases exhibited ultrastructural features typical of the LCV (arrow in **D**) or SCV (arrow in **E**) cell forms, respectively. Quantitative data were collected from at least 3 independent experiments and are presented as mean ± SD. Images of *C. burnetii* colonies were captured following 12 days of incubation and infected Vero cells were imaged 5 days post infection. Scale bars: 12.5 μm **(C)**, and 1 μm **(D,E)**.

With a conservative genome of ~ 2 Mb, encoding for 1,814 putative genes (Beare et al., 2009), *C. burnetii* appears to have a nearly intact core metabolic machinery (Seshadri et al., 2003; Omsland and Heinzen, 2011). The first axenic media designed to support replication of *C. burnetii* included a wide range of nutrients (Omsland et al., 2009, 2011) that satisfied predicted auxotrophies but also exploited the predicted capacity of *C. burnetii* to synthesize most metabolites *de novo*. To establish conditions for testing *C. burnetii* nutrient requirements, a nutritionally simple and chemically defined medium was developed. A systematic assessment of *C. burnetii* nutrient requirements revealed that the organism generates infectious progeny in a medium with a surprisingly simple nutrient composition. An axenic medium containing only 17 amino acids as organic macronutrients, Defined Acidified Citrate Medium (D-ACM), supported a 3.01 ± 0.28 log expansion in bacterial GE over 12 days, compared to 2.39 ± 0.08 log for ACCM-2, as measured by quantitative PCR (qPCR). Compared to ACCM-2, culture of *C. burnetii* in D-ACM was characterized by an extended 4-day long lag phase (Figure 2). Unlike ACCM-2 (Omsland et al., 2011), D-ACM supported development of clearly defined colonies without a soft-agarose overlay when seeded to solid medium (Figures 2B,B'). To confirm that *C. burnetii* cultivated in D-ACM remain infectious, D-ACM-expanded bacteria were used to infect cultured Vero cells and development of CCVs assessed by microscopy. A *C. burnetii* transformant containing the plasmid vector pJB-CAT (Omsland et al., 2011), which confers chloramphenicol resistance and mCherry expression, verified production of typical CCVs following culture in D-ACM (Figure 2C). Bacteria cultivated in D-ACM exhibited ultrastructural features characteristic of LCV and SCV forms, including different cell sizes (Coleman et al., 2004) in logarithmic and stationary phase, respectively (Figures 2D,E).

**Figure 3:**
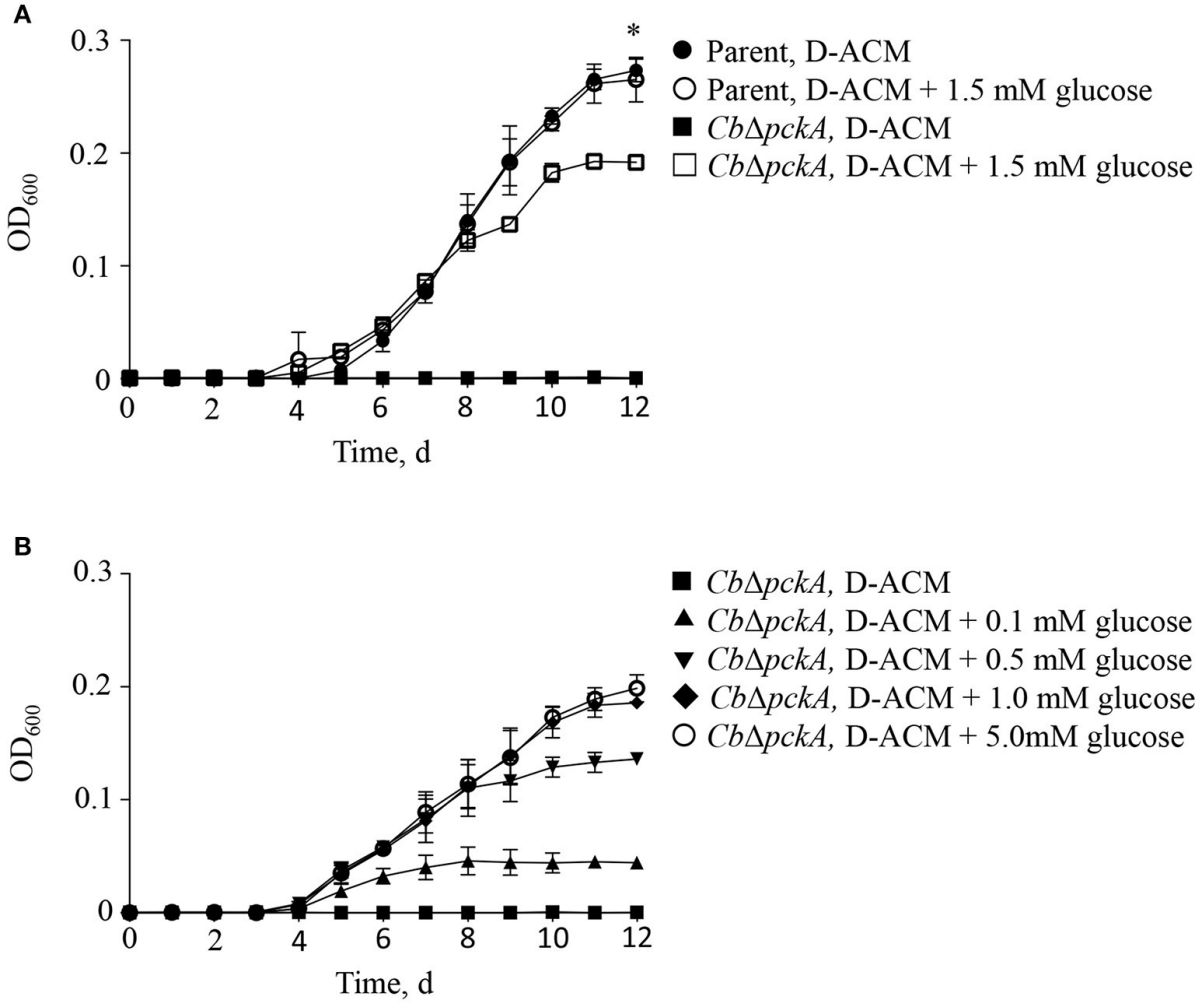
*****C. burnetii*** utilization of glucose**. The effect of glucose availability on *C. burnetii* biomass production was tested by genetically disrupting the process of gluconeogenesis. **(A)** Mutant *Cb*Δ*pckA* was unable to replicate in the presence of amino acids only. Compared to the *C. burnetii* parental strain cultivated in D-ACM over 12 d, supplementation of D-ACM with 1.5 mM glucose resulted in near complete recovery of *Cb*Δ*pckA*. Availability of glucose did not affect replication of the parental strain. **(B)** Cultivation of *Cb*Δ*pckA* in D-ACM supplemented with 0, 0.1, 0.5, 1.0, and 5.0 mM glucose showed dose-dependent utilization of the substrate. Data were collected from 3 independent experiments and are presented as mean ± SD. Asterisk indicates statistical significance between the parental strain and *Cb*Δ*pckA* (Student's *t*-test, *P* < 0.05).

The *C. burnetii* genome encodes a predicted proton-driven glucose transporter (CBU0265) (Seshadri et al., 2003) and the ability of *C. burnetii* to utilize glucose has been demonstrated biochemically (Hackstadt and Williams, 1981a). Moreover, gene expression data suggest *C. burnetii* utilizes glucose during intracellular replication (Kuley et al., 2015a). However, because *C. burnetii* does not appear to express hexokinase, the significance of glucose utilization in *C. burnetii* has been questioned (Omsland and Heinzen, 2011; Sandoz et al., 2016). The process of gluconeogenesis allows bacteria to generate core (glycolytic) metabolites from gluconeogenic substrates such as amino acids. The gene *pckA* encodes for phosphoenolpyruvate carboxykinase (PEPCK), the first committed step of gluconeogenesis. To directly determine the ability of *C. burnetii* to utilize either gluconeogenic vs. glycolytic metabolism for replication, the ability of the parental *C. burnetii* strain and a *pckA* mutant, *Cb*Δ*pckA*, to replicate in D-ACM, or D-ACM supplemented with (α-D)-glucose was measured (Figure 3). As expected, *Cb*Δ*pckA* was unable to generate biomass in non-supplemented D-ACM (i.e., provided only amino acids) but exhibited near complete growth recovery with the addition of 1.5 mM glucose, as compared to the parental strain. The parental strain showed no measurable growth difference in medium supplemented with glucose (Figure 3A), consistent with a preference for amino acids. Moreover, the response of *Cb*Δ*pckA* replication to glucose was dose-dependent (Figure 3B).

**Figure 4:**
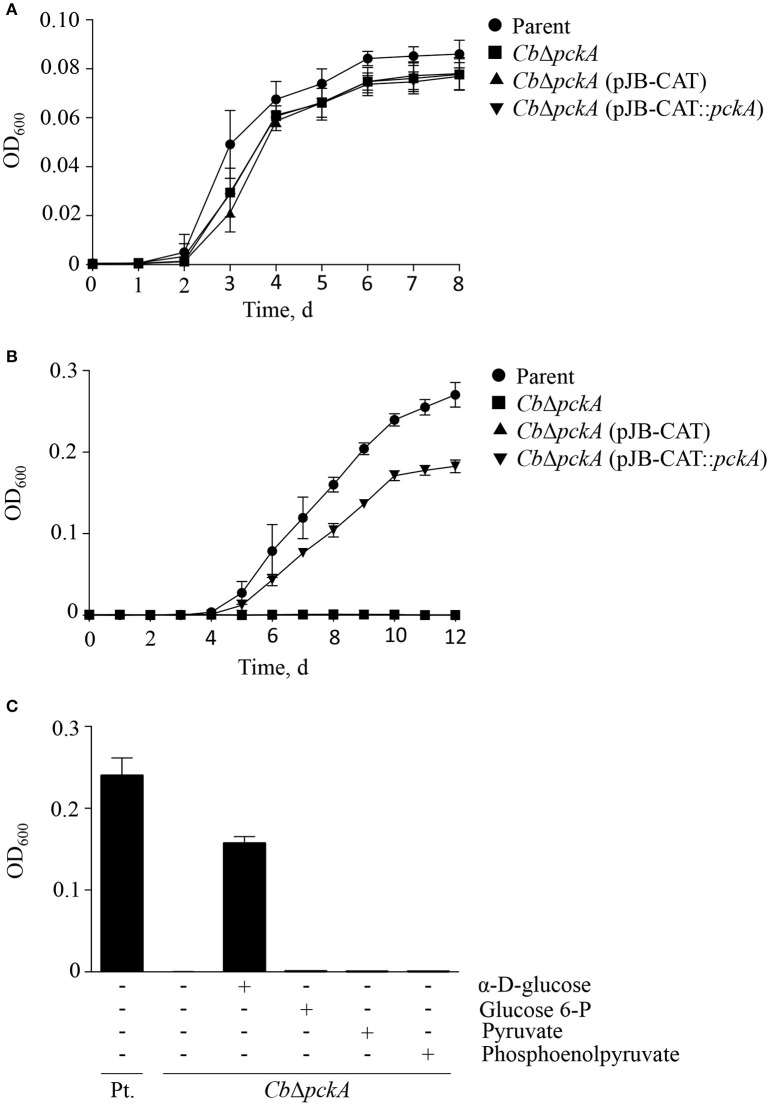
**Characterization of ***Cb***Δ***pckA*****. *Cb*Δ*pckA* was characterized to determine overall growth characteristics, metabolic deficiency and nutrient requirements. **(A)** The parental strain, *Cb*Δ*pckA, Cb*Δ*pckA* (pJB-CAT), and *Cb*Δ*pckA* (pJB-CAT::*pckA*) with *pckA* under the control of a constitutive promoter showed indistinguishable growth characteristics in ACCM-2, a medium containing both glycolytic and gluconeogenic substrates. **(B)** *Cb*Δ*pckA* and *Cb*Δ*pckA* (pJB-CAT) did not replicate on gluconeogenic substrates in plain D-ACM. Complementation of *Cb*Δ*pckA* with *pckA* restored replication. **(C)** The parental strain but not *Cb*Δ*pckA* was able to replicate in plain D-ACM. When D-ACM was supplemented with glucose, glucose 6-phosphate, pyruvate or phosphoenolpyruvate (1.5 mM), only glucose allowed chemical rescue of *Cb*Δ*pckA*. Data were collected from 3 independent experiments and are presented as mean ± SD.

In an effort to restore gluconeogenic capacity in *Cb*Δ*pckA*, a *pckA*-complemented strain [*Cb*Δ*pckA* (pJB-CAT::*pckA*)] as well as a variant carrying an empty vector [*Cb*Δ*pckA* (pJB-CAT)] were designed. Initially, strains were tested to determine whether genetic manipulation resulted in gross phenotypic changes under conditions where both glycolysis and gluconeogenesis can occur. Growth data comparing the parental strain, *Cb*Δ*pckA, Cb*Δ*pckA* (pJB-CAT::*pckA*), and *Cb*Δ*pckA* (pJB-CAT) revealed indistinguishable growth when cultivated using ACCM-2 (Figure 4A). Then, to determine metabolic capabilities, D-ACM was utilized to test phenotypes. While *Cb*Δ*pckA* was unable to replicate on gluconeogenic substrates in D-ACM, complementation with *pckA* resulted in restoration of growth (Figure 4B). Chemical rescue of *Cb*Δ*pckA* by glucose verified a metabolite deficiency consistent with defective gluconeogenesis (Figure 4C). *Cb*Δ*pckA* did not show any response to glucose 6-phosphate, pyruvate or phosphoenolpyruvate, suggesting *C. burnetii* is unable to utilize these substrates as nutrients. Although, a moderate reduction in culture yield was observed during replication of *Cb*Δ*pckA* on glucose, compared to the parental strain in plain D-ACM, the log phase replication rates (Figure 3A) were similar. This data suggests that *C. burnetii* can use glucose as a substrate via glycolytic metabolism almost as efficiently as gluconeogenesis when cultured on amino acids.

**Figure 5:**
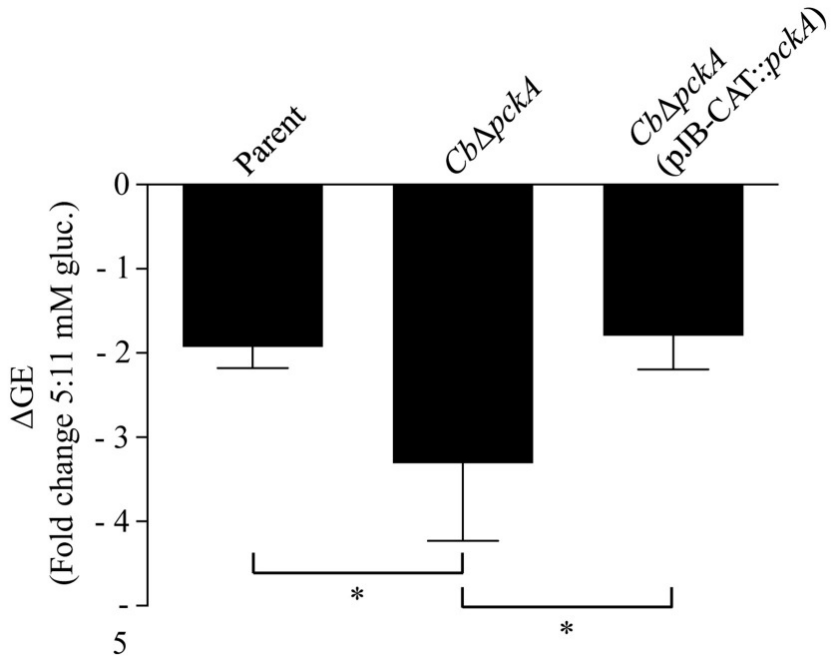
*****pckA*** promotes intracellular replication fitness under conditions of reduced glucose availability**. The ability of the parental strain, *Cb*Δ*pckA*, and *Cb*Δ*pckA* (pJB-CAT::*pckA*) were compared regarding their capacity to respond to reduced glucose availability during intracellular replication. Vero cell cultures were established under standard (11 mM) glucose conditions. Following infection, cultures were incubated in medium containing either 11 or 5 mM glucose and fold reduction in bacterial GE (ΔGE) for each strain was determined following 5 d of incubation, representing early stationary phase for the parental strain. *Cb*Δ*pckA* exhibited a moderate growth defect compared to the parental strain that was reversed upon complementation with *pckA*. Data were collected from at least 3 independent experiments and are presented as mean ± SD. Asterisks indicate statistical significance between identified samples (ANOVA, Sidak's post-test, *P* < 0.05).

The inability of *Cb*Δ*pckA* to utilize amino acids for replication combined with a dose-dependent response to glucose availability suggests that loss of gluconeogenic capacity impacts *C. burnetii* intracellular replication in a glucose-dependent manner. Potential sources of glucose for *C. burnetii* during infection include host cell constituents delivered to the CCV via autophagy, and transport of glucose into the CCV from the extracellular environment, for example via fluid phase endocytosis (Heinzen et al., 1996). To determine whether deletion of *pckA* affects intracellular replication and/or the ability of *C. burnetii* to respond to glucose availability in the context of intracellular replication, the parental strain, *Cb*Δ*pckA*, and *Cb*Δ*pckA* (pJB-CAT::*pckA*) were used to infect Vero cells maintained in medium containing different concentrations of glucose and differences in bacterial loads measured by GE analysis after 5 d of incubation (Figure 5). Deletion of *pckA* did not severely affect intracellular replication under control conditions (data not shown). However, while the parental strain showed a 1.9 ± 0.25-fold reduction in yield between normal (11 mM) and reduced (5 mM) glucose conditions, *Cb*Δ*pckA* showed a reduction of 3.3 ± 0.92-fold. Complementation with *pckA* in trans resulted in recovery of the growth defect to 1.8 ± 0.39-fold, similar to that observed for the parental strain. Overall, these data are consistent with loss of gluconeogenic capacity only resulting in a moderate glucose-dependent fitness defect during *C. burnetii* replication in Vero cells. This phenotype is indicative of efficient glucose utilization by *C. burnetii* during infection.

## Discussion

Defining the factors that govern the obligate intracellular lifestyle of bacterial pathogens such as *Chlamydia, Rickettsia*, and *Coxiella* is important for understanding their virulence, evolution and continued patho-adaptation. Here, we determined physiologically relevant physicochemical and nutritional parameters required for *C. burnetii* replication, and highlighted the ability of *C. burnetii* to replicate under limited access to organic nutrients. Based on our results, *C. burnetii* appears physiologically niche restricted to an acidified environment within respiring tissues where amino acids can satisfy macronutrient requirements. *C. burnetii* was dependent on a strict combination of physicochemical growth requirements including acidic pH, reduced O_2_ and presence of CO_2_, providing a physiological explanation for *C. burnetii* niche restriction. *C. burnetii* replication at moderately reduced temperature suggest the pathogen can adapt to replicate in arthropod vectors, as previously reported via infection of tick cells (Herrin et al., 2011).

*C. burnetii* does not have an absolute requirement for exogenous glucose for replication (Sandoz et al., 2016). However, glycolytic intermediates represent essential core metabolites and glycolytic and/or gluconeogenic capacity is necessary to generate these intermediates. Because *C. burnetii* is auxotrophic for several amino acids, experimental manipulation of amino acid availability will directly affect potential for biomass generation. By genetically disrupting *pckA*, the processes of gluconeogenesis and glycolysis were isolated biochemically and the ability of *C. burnetii* to utilize amino acids or glucose to fuel central metabolic processes could be compared under conditions of identical amino acid availability. Chemical complementation of *Cb*Δ*pckA* with glucose showed that *C. burnetii* can indeed utilize glucose to generate biomass, provided amino acid requirements are satisfied. Overall, *C. burnetii* can utilize both glycolysis and gluconeogenesis to generate essential core metabolites. This metabolic plasticity could improve replication under conditions where amino acids that fuel gluconeogenesis are available at sub-optimal levels. For example, *C. burnetii* scavenging of host amino acids during intracellular replication may deplete critical amino acids that funnel into the TCA cycle thus creating a need for glucose to fuel the core metabolic machinery. Although moderate, the growth defect observed for *Cb*Δ*pckA* during intracellular replication in Vero cells maintained under different glucose availability is consistent with a requirement for gluconeogenic capacity for optimal fitness. Moreover, even in the absence of *pckA* and thus gluconeogenic capacity, *C. burnetii* can generate essential core metabolites to support intracellular replication, likely via glycolytic metabolism. The ability of *C. burnetii* to utilize glucose during intracellular replication is consistent with findings in a recent transcriptome analysis (Kuley et al., 2015a). Deletion of *pckA* in *Mycobacterium bovis* (Liu et al., 2003), and *M. tuberculosis* (Marrero et al., 2010) results in reduced intracellular replication and virulence. The significance of *C. burnetii* glycolytic and gluconeogenic metabolism for optimal physiological fitness and maintenance of viability during infection remains to be determined.

Unlike most pathogenic bacteria that can be argued to share mechanisms for transient intracellular survival (Casadevall, 2008), bacterial obligate intracellular parasites are unique in their “physiological limitation” to only replicate within a host cell; severe niche restriction is therefore characteristic of this group of pathogens. Unlike niche restriction based on symbiosis between two or more organisms, niche restriction in intracellular pathogenic microbes is dictated by the growth requirements of the pathogen alone. Pathoadaptation, an adaptation by intracellular bacteria that improves pathogen fitness in the context of a host (Pallen and Wren, 2007), allows obligate intracellular bacteria to function more effectively within their niches. Indeed, expression of specific virulence factors is necessary for *C. burnetii* virulence. For example, expression of the pathogen's type IVB secretion system is required for intracellular replication (Beare et al., 2011; Carey et al., 2011). As demonstrated by lack of host NADPH oxidase assembly in *C. burnetii* infected neutrophils (Siemsen et al., 2009), infection by *C. burnetii* also results in modifications to host cell functions that naturally serve to prevent bacterial growth. The critical role of autophagy for *C. burnetii* intracellular replication (Gutierrez et al., 2005; Newton et al., 2014) connects the *C. burnetii* CCV to an ample supply of amino acid, a predicted nutritional requirement based on amino acid auxotrophy and genes for 18 predicted amino acid and peptide transporters in the *C. burnetii* genome (Seshadri et al., 2003). The effector protein Cig2, secreted via the *C. burnetii* type IVB secretion system, promotes interactions between the CCV and host autophagosomes (Newton et al., 2014). Thus, Cig2 represents a molecular mechanism used by *C. burnetii* to connect its intracellular niche to a nutrient recycling system of the host cell.

Unlike determinants of niche restriction that, for example, limit *C. burnetii* to replicate in a moderately acidic environment, the aforementioned pathoadaptive traits allow *C. burnetii* to replicate with optimized fitness within the CCV. Based on predicted auxotrophy, a requirement for host cell-derived amino acids in *C. burnetii* replication is not surprising. As shown with regulation of developmental transitions by threonine availability in *L. pneumophila* (Sauer et al., 2005), and a critical role for asparagine in *Francisella tularensis* intracellular replication and virulence (Gesbert et al., 2014), the ability to sense and respond to specific amino acids is a physiological adaptation of several intracellular pathogens. The significance of specific amino acids in *C. burnetii* physiology has yet to be determined.

Organisms with physicochemical growth requirements similar to those observed for *C. burnetii* include *Campylobacter jejuni* and *Helicobacter pylori*, both of which replicate extracellularly. Like *C. burnetii, H. pylori* responds to O_2_ and CO_2_ tension, as well as moderately acidic pH. However, unlike *C. burnetii, H. pylori* has a wider range of permissive growth conditions, including robust replication over a pH range from 4.5 to 8 (Kangatharalingam and Amy, 1994). Therefore, a major difference between these organisms is the inability of *C. burnetii* to effectively adjust to sub-optimal environments. As shown in Figure 1, a change in pH from 4.75 to 5.0 essentially resulted in loss of cell division. It should be noted that *C. burnetii* is likely capable of adapting to a wider range of conditions and that intermediate levels of replication could be achieved under these conditions. For instance, the physiological basis for microaerophily is potentially related to oxidative stress sensitivity and/or dependence on the reduced form of a nutrient. Moreover, as seen with *C. jejuni* (Bolton and Coates, 1983) and *H. pylori* (Bury-Moné et al., 2006), the effects of O_2_ and CO_2_ tension can be inter-related. The mechanistic basis for physiological adaptation to reduced O_2_ and elevated CO_2_ is unclear. Nonetheless, for *C. burnetii*, replication is positively affected by a combination of moderately acidic pH, reduced oxygen availability, and presence of CO_2_; these parameters can be traced back to respiring tissues, such as the spleen, colonized in animals infected by *C. burnetii* (Russell-Lodrigue et al., 2009).

Like the chemically defined medium ACCM-D (Sandoz et al., 2016), D-ACM shows that *C. burnetii* can replicate with amino acids as the only macronutrients. Because nutrient compositions are defined, both media can be employed to study *C. burnetii* metabolic capabilities. However, optimal nutrient conditions can mask the effect of individual nutrients on bacterial growth. Therefore, D-ACM was designed to be both nutritionally simple (few components) and limited (supporting robust yet sub-optimal replication) to facilitate the study of specific nutrients on *C. burnetii* physiology. The medium contains macronutrients at equimolar concentrations to facilitate analysis of the relative significance of specific substrates. D-ACM contains 17 amino acids that together support robust growth of *C. burnetii* using both liquid and solid media. Due to the reported role of glutamate as a preferred carbon source for *C. burnetii* (Hackstadt and Williams, 1981b), D-ACM contains glutamate but not glutamine which can be synthesized from glutamate. Supplementation of D-ACM with glutamine had no measurable effect on replication while the presence of serine resulted in a moderate reduction in growth (data not shown).

Replication of *L. pneumophila*, an organism phylogenetically related to *C. burnetii*, has been achieved using a medium containing only 9 amino acids (Tesh and Miller, 1981), suggesting amino acids alone can support replication of this organism. The ability to expand and sub-culture *C. burnetii* in D-ACM prepared from analytical standard grade amino acids suggests that amino acids alone satisfy *C. burnetii* nutrient requirements (data not shown). Indeed, *C. burnetii* is likely capable of synthesizing several micronutrients expected to be critical for replication. For example, pathways for biosynthesis of biotin and folic acid appear to be intact (Seshadri et al., 2003). Also, a homolog of the gene *panG*, a novel ketopantoate reductase shown to have activity complementary to *panE* (Miller et al., 2013), completes the canonical pathway for pantothenate biosynthesis in *C. burnetii*. The chemical simplicity of D-ACM will facilitate biochemical analysis of *C. burnetii* micronutrient requirements.

### Pathoadaptive consequences of intracellular niche restriction in *C. burnetii*

Severe niche restriction, as observed in bacterial obligate intracellular parasites, places unique evolutionary pressures upon a pathogen. Because eukaryotic cells depend on physiological homeostasis for viability, the relative stability of an intracellular niche reduces overall evolutionary pressure on a parasite. Under conditions where evolutionary pressure is of low intensity and diversity, mutations are expected to accumulate in a wide range of genes whose functions are not essential. In bacterial obligate intracellular parasites this is in part reflected by a large number of pseudogenes (Beare et al., 2009) and, over time, a continual streamlining of the core genome. Compared to species of the genera *Rickettsia* and *Chlamydia* with genomes of ~1 Mb, *C. burnetii* can afford to further streamline its genome. Assuming genome reduction in *C. burnetii* is a work in progress, initial loss of genes associated with synthesis of amino acids may be a result of the high cost of amino acid biosynthesis. Moreover, and as mentioned for *C. burnetii*, parasitic bacteria naturally draw on metabolite pools maintained by the host. Thus, *C. burnetii* has potentially adapted to improve metabolic fitness in a vacuole connected to the host autophagic machinery (Gutierrez et al., 2005; Kohler and Roy, 2015), which recycles these essential molecules. Because *C. burnetii* can utilize glucose, the pathogen has the theoretical capacity to synthesize essential core metabolites via glycolysis, the reductive branch of the pentose phosphate pathway, and the TCA cycle. However, amino acid auxotrophy (Seshadri et al., 2003; Sandoz et al., 2016) dictates that *C. burnetii* replication can only take place in a niche containing amino acids.

Host cells invaded by parasitic bacteria can respond to infection by innate immune mechanisms that prevent pathogen access to specific nutrients. An example of this is the enzyme-dependent depletion of host tryptophan pools in cells infected with *C. trachomatis* (Beatty et al., 1994). Such host responses provide a selective pressure to the invading parasite, where failure of the intracellular parasite to adapt precludes the fitness required for the pathogen to replicate in this niche. The ability to scavenge sufficient nutrients from the host ultimately regulates pathoadaption of intracellular bacterial parasites with complex auxotrophy such as inability to synthesize multiple amino acids. Little evidence is available on where in this tug-of-war *C. burnetii* exists with its host; however, data generated using host cell-free culture tools has revealed signs of nutritional constraints that may influence *C. burnetii* pathoadaptation.

Several bacterial intracellular parasites, including *C. burnetii* (Hackstadt and Williams, 1981b), appear to use amino acids as the preferred carbon and energy source. *L. pneumophila* induces rapid changes in host amino acid pools upon infection to support pathogen replication (Bruckert et al., 2014). Amino acid auxotrophy in *C. burnetii* (Seshadri et al., 2003; Sandoz et al., 2016) is consistent with amino acid acquisition and metabolism being essential aspects of *C. burnetii* physiology during intracellular replication. Continued optimization of amino acid acquisition and metabolism are likely areas of continued pathoadaptation in *C. burnetii*. Analysis of *C. burnetii* mutants with defects in amino acid metabolism and pathways related to replication with amino acids as the primary carbon and energy sources (e.g., the TCA cycle, gluconeogenesis, and the pentose phosphate pathway) will be necessary to identify metabolic requirements for *C. burnetii* during intracellular replication. Data generated using the parental strain and a *pckA* mutant of *C. burnetii* NMII under defined nutrient conditions allowed conclusive demonstration that *C. burnetii* can utilize both amino acids and glucose to support replication. Under conditions of ample amino acid availability, glucose availability did not further enhance replication.

### Unraveling the molecular basis for *C. burnetii* niche restriction

**Figure 6:**
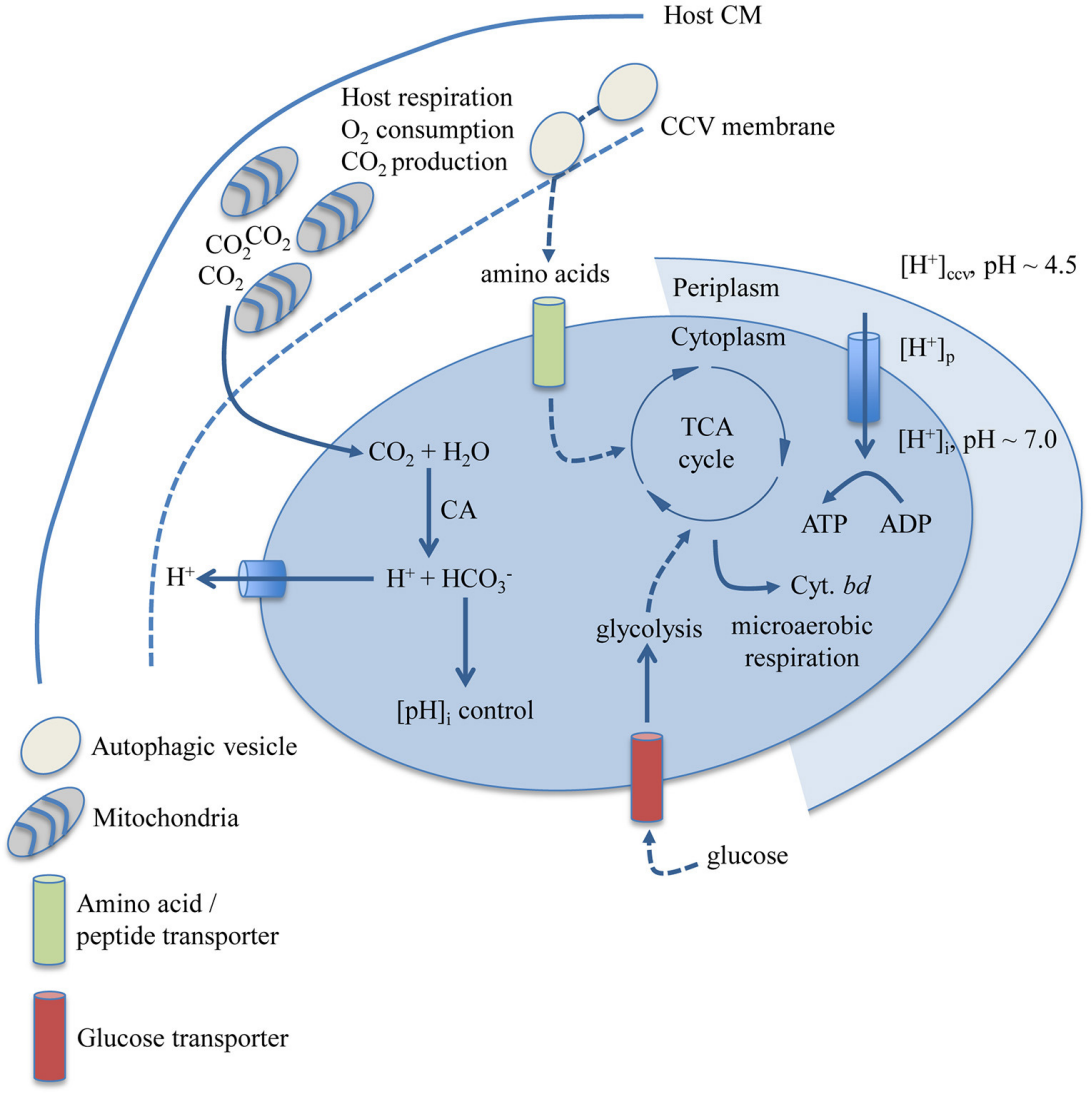
**Biological determinants of niche restriction in ***C. burnetii*****. Nutritional and physicochemical variables determined to impact *C. burnetii* replication and biochemical pathways and enzymatic reactions with documented involvement in these processes are depicted. *C. burnetii* can utilize both amino acids and glucose, the latter likely transported by a proton-driven glucose transporter encoded by CBU0265. The cytochrome *bd* terminal oxidase is predicted to allow *C. burnetii* respiration in a microaerobic environment. The subcellular localization of the *C. burnetii* CA (CBU0139), possibly involved in CO_2_-dependent regulation of *C. burnetii* cytoplasmic pH, has not been determined. CM, cytoplasmic membrane; CCV, *Coxiella* Containing Vacuole; CA, carbonic anhydrase; Cyt., cytochrome.

This study makes an argument for how gross physicochemical and nutritional factors affecting *C. burnetii* replication can explain why *C. burnetii* appears restricted to a specific niche. Figure 6 is a graphical representation of how the findings presented herein may correlate with determinants of metabolic capacity in *C. burnetii*. Moderately acidic pH likely contributes to establishment of a proton motive force and metabolite transport in *C. burnetii* (Hackstadt, 1983; Hackstadt and Williams, 1983). The *C. burnetii* carbonic anhydrase (CA, CBU0139), an enzyme with functions in maintenance of intracellular pH and response to acid stress (Bury-Moné et al., 2008), is logically connected to the observed requirement for CO_2_ in an acidic environment such as the CCV. Physiological dependence on CO_2_ could also be related to the activity of enzymes that require CO_2_ or bicarbonate (${\text{HCO}}_{3}^{-}$), including carbamoyl phosphate synthase (Anderson and Meister, 1965) (CBU1281/CBU1282), and biotin carboxylase (Attwood and Wallace, 2002) (CBU1726). *Helicobacter pylori* dependence on CO_2_ has been linked to activity of acetyl-CoA carboxylase (Burns et al., 1995), an enzyme complex containing biotin carboxylase. Carbamoyl phosphate was early implicated in *C. burnetii* glucose utilization (Paretsky et al., 1962), and a potential pathway for glucose utilization via carbamoyl phosphate synthase and glucose-6 phosphatase has been suggested based on the *C. burnetii* genome sequence (Omsland and Heinzen, 2011). The branched respiratory chain of *C. burnetii* containing genes for expression of the cytochrome *bd* terminal oxidase (CydA/B) with high affinity for O_2_ originally motivated analysis of *C. burnetii* requirements for replication under microaerobic conditions (Omsland et al., 2009).

Numerous isolates of *C. burnetii* have been recovered and variable replication in the host cell-free medium ACCM-2 (Kersh et al., 2011; Omsland et al., 2011; Kuley et al., 2015b) suggests that *C. burnetii* metabolic capacity varies between isolates. Therefore, the ability of *C. burnetii* NMII to replicate in a medium that only contains amino acids may not reflect the nutrient requirements of all *Coxiella* isolates. Although, *Coxiella* shows isolate-specific nutrient requirements, all isolates replicate within intracellular vacuoles and thus appear to exhibit the same degree of niche restriction. The possibility that the physiological basis for niche restriction among *C. burnetii* isolates could vary cannot be excluded. However, because the isolates that have been cultured under controlled host cell-free conditions were cultured in ACCM-2 at moderately acidic pH under reduced O_2_ and elevated CO_2_ tension (Kersh et al., 2011, 2016; Omsland et al., 2011; Kuley et al., 2015b), these physicochemical parameters currently represent common denominators for axenic culturability. The extent to which these physicochemical parameters affect replication, and how nutrient availability and isolate-specific metabolic plasticity factor in to niche restriction in the genus *Coxiella* remains to be determined.

This study largely relied on host cell-free culture tools necessary to isolate *C. burnetii* from the host to determine how specific physicochemical and nutritional factors affect *C. burnetii* only. The *C. burnetii* Dot/Icm type IVB secretion system, a virulence determinant not required for replication under host cell-free conditions, is critical for CCV biogenesis and pathogen intracellular replication (Beare et al., 2011; Carey et al., 2011). Ultimately, a combination of host cell-free culture tools and models for *C. burnetii* intracellular replication will be required to fully understand parameters of niche restriction in *C. burnetii*. The axenic culture techniques described in this study will facilitate the use of several fundamental bacteriological techniques, including clonal isolation and colony forming unit assays, in these efforts.

## Author contributions

EVE, HY, SES, and AO: produced and analyzed data, and wrote the manuscript. AO: designed the study.

### Conflict of interest statement

The authors declare that the research was conducted in the absence of any commercial or financial relationships that could be construed as a potential conflict of interest.
