# Supplementary material for: Physicochemical and Nutritional Requirements for Axenic Replication Suggest Physiological Basis for Coxiella burnetii Niche Restriction
Source: Front Cell Infect Microbiol. 2017 May 31;7:190. doi: 10.3389/fcimb.2017.00190 (PMC5449765; doi:10.3389/fcimb.2017.00190)
Supplement: Supplementary file 1 [file Table1.docx]

Table S1. D-ACM recipe

| Component | Formula / FW^a^ | mg / 200 ml | Final concentration |
| --- | --- | --- | --- |
| 2X Basal Buffer |  |  |  |
| Citric acid | C_6_H_8_O_7_ | 513.6 | 13.4 mM |
| Sodium citrate | Na_3_C_6_H_5_O_7_x2H_2_O | 948.0 | 16.1 mM |
| Potassium phosphate | KH_2_PO_4_ | 100.0 | 3.7 mM |
| Magnesium chloride | MgCl_2_x6H_2_O | 40.0 | 1.0 mM |
| Sodium chloride | NaCl | 1456.0 | 124.7 mM |
| Calcium chloride | CaCl_2_x2H_2_O | 2.6 | 90.0 μM |
| Iron sulfate | FeSO_4_x7H_2_O | 0.56 | 10.0 μM |
|  |  |  |  |
| Amino Acids |  |  |  |
| Methionine^b^ | 149.2 | 44.8 | 1.5 mM |
| Arginine^b^ | 210.6 | 63.2 | 1.5 mM |
| Glycine^b^ | 75.1 | 22.5 | 1.5 mM |
| Cysteine^b^ | 157.6 | 47.3 | 1.5 mM |
| Proline^b^ | 115.1 | 34.5 | 1.5 mM |
| Lysine^b^ | 182.7 | 54.8 | 1.5 mM |
| Isoleucine^b^ | 131.2 | 39.4 | 1.5 mM |
| Tyrosine^c^ | 181.2 | 54.4 | 1.5 mM |
| Alanine^d^ | 89.1 | 26.7 | 1.5 mM |
| Valine^d^ | 117.2 | 35.2 | 1.5 mM |
| Asparagine^d^ | 132.1 | 39.6 | 1.5 mM |
| Threonine^d^ | 119.1 | 35.7 | 1.5 mM |
| Histidine^d^ | 209.6 | 62.9 | 1.5 mM |
| Phenylalanine^d^ | 165.2 | 49.6 | 1.5 mM |
| Leucine^d^ | 131.2 | 39.4 | 1.5 mM |
| Tryptophan^d^ | 204.2 | 61.3 | 1.5 mM |
| Glutamic acid^d^ | 147.1 | 44.1 | 1.5 mM |
|  |  |  |  |
| Supplement |  |  |  |
| Methyl-β-cyclodextrin | - | 200 | 1 mg/ml |

^a^Formula Weight shown for amino acids.

^b^Dissolved in dH_2_O.

^c^Dissolved in 1N HCl.

^d^Dissolved in 2X Basal Buffer.
