# Supplementary figures and images for: Physicochemical and Nutritional Requirements for Axenic Replication Suggest Physiological Basis for Coxiella burnetii Niche Restriction
Source: Front Cell Infect Microbiol. 2017 May 31;7:190. doi: 10.3389/fcimb.2017.00190 (PMC5449765; doi:10.3389/fcimb.2017.00190)

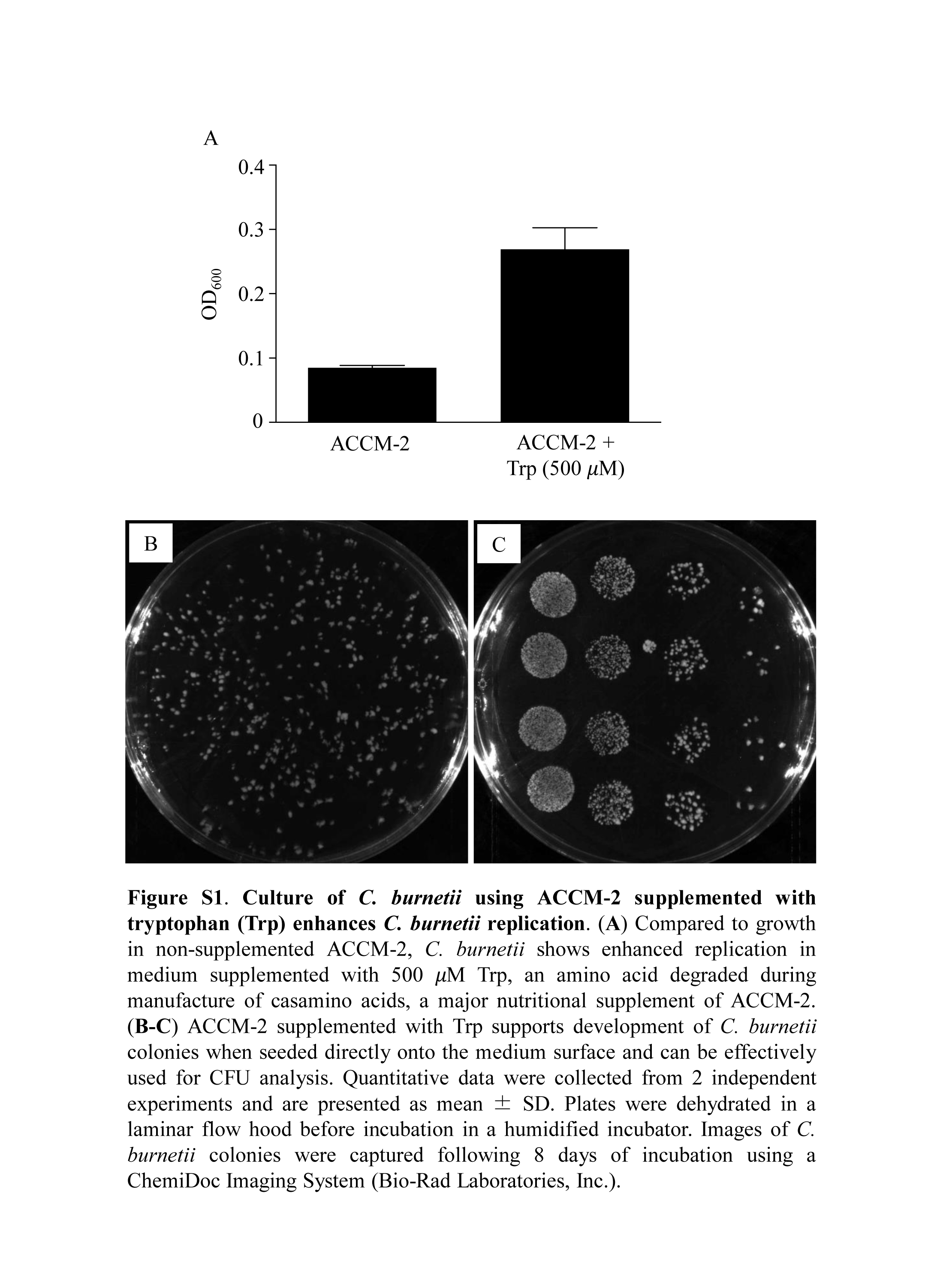

Supplement: Supplementary file 2 [file Image1.jpg]

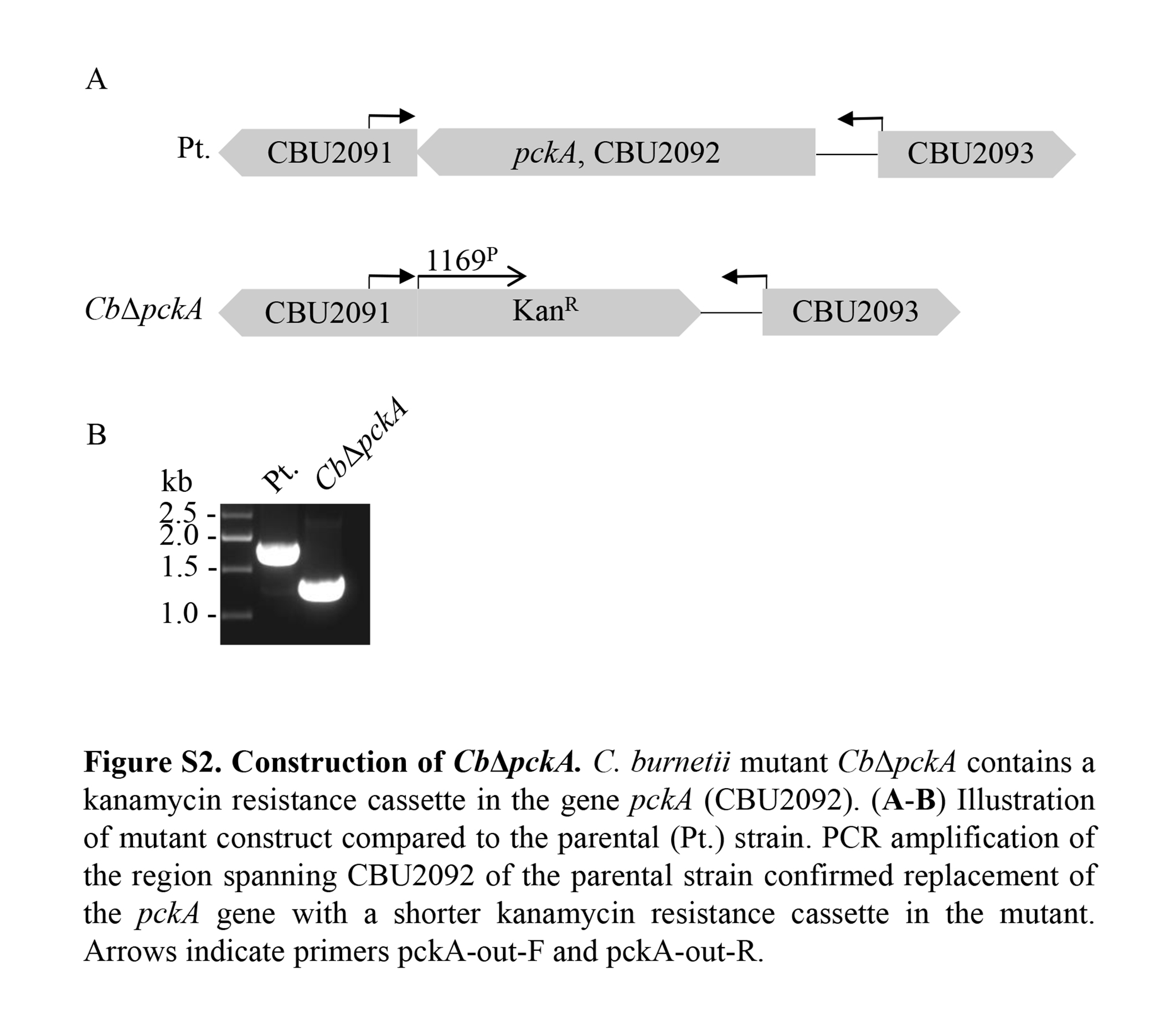

Supplement: Supplementary file 3 [file Image2.JPEG]
